# Nutritional, environmental and economic implications of children plate waste at school: a comparison between two Italian case studies

**DOI:** 10.1017/S136898002400034X

**Published:** 2024-02-16

**Authors:** Beatrice Biasini, Michele Donati, Alice Rosi, Francesca Giopp, Irena Colić Barić, Martina Bituh, Ružica Brečić, Mary Brennan, Ana Ilić, Steve Quarrie, Maysara Sayed, Angela Tregear, Davide Menozzi, Francesca Scazzina

**Affiliations:** 1 Department of Food and Drug, University of Parma, Parma, Italy; 2 Department of Chemistry, Life Sciences and Environmental Sustainability, University of Parma, Parco Area delle Scienze, 33/a, Parma 43124, Italy; 3 Department of Food Quality Control, Faculty of Food Technology and Biotechnology, University of Zagreb, Zagreb, Croatia; 4 Marketing Department, Faculty of Economics and Business, University of Zagreb, Zagreb, Croatia; 5 University of Edinburgh Business School, Edinburgh, UK; 6 European Training Academy, Belgrade, Serbia

**Keywords:** Plate waste, School meal, Nutritional adequacy, Nutritional loss, Carbon footprint, Economic impact

## Abstract

**Objective:**

This study aims at comparing two Italian case studies in relation to schoolchildren’s plate waste and its implications, in terms of nutritional loss, economic cost and carbon footprint.

**Design:**

Plate waste was collected through an aggregate selective weighting method for 39 d.

**Setting:**

Children from the first to the fifth grade from four primary schools, two in each case study (Parma and Lucca), were involved.

**Results:**

With respect to the served food, in Parma, the plate waste percentage was lower than in Lucca (*P* < 0·001). Fruit and side dishes were highly wasted, mostly in Lucca (>50 %). The energy loss of the lunch meals accounted for 26 % (Parma) and 36 % (Lucca). Among nutrients, dietary fibre, folate and vitamin C, Ca and K were lost at most (26–45 %). Overall, after adjusting for plate waste data, most of the lunch menus fell below the national recommendations for energy (50 %, Parma; 79 %, Lucca) and nutrients, particularly for fat (85 %, Parma; 89 %, Lucca). Plate waste was responsible for 19 % (Parma) and 28 % (Lucca) of the carbon footprint associated with the food supplied by the catering service, with starchy food being the most important contributor (52 %, Parma; 47 %, Lucca). Overall, the average cost of plate waste was 1·8 €/kg (Parma) and 2·7 €/kg (Lucca), accounting respectively for 4 % and 10 % of the meal full price.

**Conclusion:**

A re-planning of the school meals service organisation and priorities is needed to decrease the inefficiency of the current system and reduce food waste and its negative consequences.

School food procurement has the pivotal role of offering students energetically and nutritionally adequate meals to support their growth and development, school performance, and to lay foundation for healthy and balanced dietary habits into adulthood^(1)^. Beside social and health functions, collective catering can foster the environmental protection by encouraging sustainable food production methods. In Europe, recent policies, such as Green Public Procurement^(2)^ and Directive 2014/24^(3)^, suggest how to reduce the environmental burden of supply chains in public institutions and recommend improving the quality of public services through the achievement of economic, environmental and societal benefits. Accordingly, instead of applying the sole price/cost criterion, the best price/quality ratio should orient contract awarding in European countries^(3)^. National and regional authorities have made efforts in this direction by encouraging public food procurement to offer local food, traditional products or products coming from sustainable production methods (e.g. organic agriculture). Based on the awarding criteria and the analysis of the contract tenders stipulated between the catering services and the public authorities (e.g. municipalities), different food procurement models can be defined. For example, a food procurement can be defined as local and/or organic if it is primarily based on local and/or organic food products, while a model can be defined as ‘low cost’ if no quality requirements are specified in the contract which relies only on the most economically advantageous offer.

In Italy, the public administration promotes the improvement of school catering service sustainability: by designing healthy and balanced meals compliant with the national dietary guidelines^(4)^; by promoting seasonal and locally sourced food and organic products (with a provision of organic fruit, vegetables, legumes, cereals and bovine meat set to at least 50 % by weight); by encouraging the consumption of low-cost protein sources, such as legumes, alternative fish species and meat cuts; and by including recipes prepared with edible parts of fruit and vegetables usually discarded^(5)^. Recommendations to minimise food waste are also included. Among these, relevant measures are the monitoring of food surplus and waste with a standard procedure, the identifications of the main related critical issues, and the development of educational and awareness-raising programmes on food waste involving children and their families^(6)^. Food waste quantification and reduction strategies are therefore crucial in the public procurement sector, which is directly implicated in the promotion of sustainable practices^(7)^, consistently with the national and international policies and priorities.

It is worth noting that, globally, in 2019, food waste has been estimated to be approximately 931 million tonnes, an amount mainly ascribable to households (61 %) followed by food service (26 %) and retail (13 %), corresponding to about one-sixth (17 %) of the food globally produced^(8)^.

In 2015, the UN launched an international call to halve the global food waste per capita, both at the retail and at the consumer level, and recommended to target food losses originating during the production phase and along the supply chain by addressing substantial efforts in prevention, reduction, recycling and reuse activities^(9)^. Food waste is responsible for negative externalities in multiple dimensions. It causes an additional use of natural resources such as land, water, chemicals and energy that could be mitigated by enhancing virtuous management practices and strategies to prevent it^(10)^. According to the estimates, the food wasted at the retail and consumption level accounts for 9 % of greenhouse gas emissions (GHGe) generated by food systems^(11)^, contributing to climate change.

Among the environmental indicators, the Global Warming Potential, also referred to GHGe (kg CO_2_eq), is the most considered in the studies referring to meals served in schools^(12–15)^. A sustainability assessment tool allowing the evaluation of food impact on biodiversity has been proposed for catering companies by considering concrete targets defined per meal^(16)^. Some studies^(17–20)^ considered the impact of observed or supposed food waste scenarios at the consumption phase. However, evidence for the school sector is limited^(18,20)^.

In parallel to the environmental dimension, the global economic loss caused by food loss and waste is estimated to amount to $940 billion annually, $218 billion of which is ascribable to the USA^(21)^. In Europe, the annual generation of about 88 million tonnes of food waste (i.e. 174 kg/per capita) is associated with an estimated cost of 143 billion euros^(22)^. In the context of meals served in schools, the economic loss of plate waste has been reported by a previous study involving middle schools in Boston, where about 26 % of the total food budget was annually discarded by students at lunch^(23)^.

In the school setting, due to the difference between the amount of food planned to be consumed by children and their actual intake, food waste can exert considerable nutritional losses. For this reason, to minimise food waste in the school canteens, its quantification, analysis and monitoring are paramount^(24)^. Plate waste, defined as the quantity or proportion of food served to people but then discarded by people, could be used to estimate food intake and the efficacy of interventions developed to strengthen healthy eating behaviours at schools^(25,26)^.

This study, conducted within the framework of the Strength2Food European Project – funded by Horizon 2020 research and innovation programme (grant agreement no 678024) – is aimed at analysing and comparing two case studies in Italy in relation to children’s plate waste, and its nutritional, environmental, and economic impact. The two case studies are represented by a sample of primary schools located in the municipality of Parma (Emilia-Romagna region) and the municipality of Lucca (Tuscany region). The investigation follows a previous work in which the two municipalities have been presented and evaluated together with other eight case studies across Europe to assess the sustainability impact of different models of public procurement and discuss the actions and strategies that are more likely to address multiple sustainability outcomes^(27)^. As previously described^(27,28)^ and summarised in see online supplementary material, Supplemental Table S1, Parma and Lucca are characterised by two different food procurement models, defined respectively as local-organic (LOC-ORG) and organic (ORG).

By selecting two procurement models with a different share of local/traditional products in the meal offers, we expect to potentially find different plate waste percentages in consideration of the role of food neophobia and picky eating as crucial determinants of food rejection in children^(29)^. Therefore, the LOC-ORG model is expected to be linked to fewer children’s plate waste because of less opportunity for neophobia.

## Methods

### Case study description

LOC-ORG and ORG cases are comparable both considering the territory and food culture and traditions. For the study, a total of four primary schools, two in each municipality, have been selected by applying the following criteria: the presence of at least 100 children attending the schools and signing up for the school catering service; the model followed to prepare and distribute meals (i.e. from a central or an internal kitchen). The distance between the schools and the cooking centre was additionally considered in the ORG case, where school menu preparation was only external, contrarily to LOC-ORG case where meals were prepared in on-site or off-site kitchen, depending on the school facilities. The profile of the selected primary schools is provided in the Supplementary File (see online supplementary material, Supplemental Table S2).

In both case studies, lunch meals are designed and approved by municipal dieticians. The schools offer a daily single-option meal represented by the standard menu or a different one designed for special diets in case of allergy, celiac disease, religious reasons or specific requests. Parents have the responsibility of the menu type selection at the beginning of the school year or during it in case of contextual illnesses that can drive the selection of a meal in white. Students are personally served by the catering staff which distributes meals typically composed by starchy-based first course (i.e. cereal or cereal-derived products such as pasta), protein-based second course (i.e. eggs, meat, fish, legumes and cheese), vegetables as side dish, bread and fruit. Dessert is present only for special occasions (e.g. Christmas) in LOC-ORG case, while it is served once a week as substitute of fruit in ORG case. Children are supposed to eat all the food offered to them; they can eventually ask for a slight modification of the standard portion to be served based on their requests.

Due to the numerosity of children, the school lunch was offered in both case studies in two waves of 30 min each where students of mixed grades are served. In the LOC-ORG case, the school menu follows a four week-cycle differentiated across the four seasons, while in the ORG case it runs on a seven/eight week-cycle and differs in autumn-winter from that offered in spring-summer. This means that, within each seasonal period, the menus are identically repeated after four weeks and after seven/eight weeks, respectively.

### Data collection

Seasonal school lunch menus and normative provisions were respectively obtained from the City Council and the local manager of the central school catering services. Two weeks (one in winter 2017 and one in spring 2018) were selected in each school. Plate waste, referring to the edible fraction of served food discarded by children, was collected from all children (from the first to the fifth grade) in the school canteens, excluding those served with menus for special diets. An aggregate selective plate waste method^(30)^ was applied, collecting waste distinguishing seven food categories: starchy food; bread; protein-based dishes; vegetables; fruits; desserts; and ‘other’. The latter included dishes characterised by a comparable content of starchy and protein-based food (e.g. pizza). For each dish, the average weight of the edible served food was calculated from three servings offered at the beginning of each waves. The weight of the average servings and the collected food waste were assessed using electronic weighing scales (e.g. Parcel Digital Weighing Scale 30 kg, division: 1 g, 9901, Eva Collection).

### Data analysis

For each dish, the served food amount (g) was calculated as the average serving of edible food (g) multiplied by the number of the served children. The total plate waste (kg) and served food (kg) were obtained respectively as the sum of the food waste (kg) and as the sum of the served food for every food category for the two schools in each case study, and across both data collection weeks. The percentage of food waste for every food category was computed as the ratio between the total edible plate waste (kg) referred to each food category and the total amount of the food categories (kg) served to children. Finally, the plate waste as total and by food category (kg) was divided by the number of served children to estimate the waste per child (g). By subtracting this quantity to the average serving of edible food, the food intake per child was estimated.

Energy and nutritive values per dish planned to be served were calculated using the national composition database for epidemiological studies in Italy^(31)^. The energy and nutrient contents of each food item were summed to obtain the energy and nutritional profile of the menus. By subtracting the energy and nutrient content of plate waste to those calculated for the dishes planned to be served, an estimation of the actual energy and nutrient intakes was provided. The energy and nutritional composition of the meals as planned to be served and consumed was evaluated in comparison to the national guidelines for school lunch (being the latter depicted in see online supplementary material, Supplemental Table S3).

The environmental impact of plate waste was estimated in terms of GHGe associated with the food production and food waste management by the school meal services in the two case studies. The applied emissions factors were retrieved from a multitude of sources^(32–35)^. Specifically, the emissions factors applied to food waste follows the approach proposed by Moult and colleagues^(36)^. By multiplying the average emissions factor by the total volumes of waste collected for each food categories, the total production- and transport-related embodied carbon emissions for single food categories were estimated for both the cases. To estimate the total GHGe of the plate waste collected in the two case studies, the contribution of waste transportation and disposal method was also considered and summed to get total and specific embodied carbon emissions due to the production, transportation and waste disposal activities.

To estimate the economic loss linked to the collected plate waste, an average cost per kg of waste per food category was computed by dividing the total supply budget associated with the sampled menus by the volumes of specific items procured within each category, in proportion to each other. Specifically, the estimate of average cost per kg for single food waste categories was made through the average annual market price of every food item retrieved from the statistics provided by the national Institute of Agri-food Market Services (ISMEA). The total cost of food category waste was then summed to derive an estimate of the total cost of plate waste for the two cases.

### Statistical analysis

The normality of data distribution was explored using the Kolmogorov–Smirnov test. According to data distribution, comparisons between the two groups (LOC-ORG *v*. ORG) were tested using the Student’s *t*-test or the Mann–Whitney *U* test. Data are described as median (interquartile range) or as mean and standard deviation if data followed a non-normal or normal distribution, respectively. The statistical analysis was performed using SPSS 28.0 software (SPSS Inc.), keeping the significance at *P* < 0·05.

## Results

### Plate waste

Although in the LOC-ORG case 6196 more dishes were collected and 667 more kg of food were served, the total amount of plate waste reported here across the schools and seasons weighed 11 kg less than the counterpart (Table 1). Accordingly, the median daily plate waste corresponded to 27·3 kg and 28·3 kg in LOC-ORG and ORG model, respectively.

**Table 1:** Number of dishes, quantity of served food and waste, including the waste per d, reported as total values and by food categories per food procurement model

Food categories	Total dishes (*n*)		Total served food (kg)		Total plate waste (kg)		Plate waste/d (kg)			
	LOC-ORG	ORG	LOC-ORG	ORG	LOC-ORG	ORG	LOC-ORG		ORG	
	Total value		Total value		Total value		Median	IR	Median	IR
Bread	3988	2677	144·0	81·7	53·6	34·1	2·4	1·9–3·4	2·0	1·7–2·3
Starchy food	3526	3331	813·2	603·4	162·6	191·5	7·8	4·9–12·7	9·9	7·5–12·7
Protein dish	3471	2523	223·4	176·2	39·5	60·2	1·7	1·1–3·1	2·6	1·8–5·6
Vegetables	3979	1453	267·9	131·1	98·7	68·1	4·5	4·1–6·2	5·1	3·4–7·4
Fruit	4134	2304	539·3	338·8	163·6	180·8	6·1	4·5–11·1	11·8	7·3–17·0
Other	387	311	115·5	28·2	22·6	3·3	11·3	10·5–12·1	1·6	1·6–1·7
Dessert	–	690	–	76·7	–	13·7	–	–	1·8	1·2–4·0
Total	19 485	13 289	2103·2	1436·2	540·6	551·8	27·3	22·1–30·2	28·3	24·8–33·9

LOC-ORG, local-organic; ORG, organic.

Despite the small difference in terms of absolute values, the share of total plate waste was different between the two case studies (*P* < 0·001), corresponding to a median of 23·7 % for the LOC-ORG model and 41·5 % for the ORG model (Table 2). The same trend can be observed by considering the single food categories. Proportions of waste in four of these were higher in the ORG case than in the counterpart (*P* < 0·01). Similarly, significant differences were obtained in the waste per child for total (*P* < 0·001) and single food categories (*P* < 0·01), excepting bread and vegetables. In the ORG case, the waste of fruit and vegetables exceeded 50 % of the serving, while a low proportion of the ‘other’ dishes was wasted (11·8 %). Therefore, the median pupils’ uptake of plant-based food (i.e. fruit and vegetables) was less than the half of the average serving size served.

**Table 2:** Serving size, waste percentage with respect to the served food and waste per child expressed as total daily values and by food categories for LOC-ORG (*n* 20) and ORG model (*n* 19)

		LOC-ORG		ORG		*P*-value
		Median	IR	Median	IR	
Serving size (g)	Bread	40·6	25·3–48·5	30·2	26·3–32·3	0·133
	Starchy food	240·9	194·7–265·4	211·0	192·5–225·9	0·118
	Protein dish	55·7	40·0–93·0	60·8	51·3–76·3	0·503
	Vegetables	52·9	43·9–86·1	57·5	50·2–78·2	0·870
	Fruit	125·9	120·0–133·9	151·8	128·0–175·0	0·033
	Other	293·7	261·0–326·4	93·0	80·6–105·4	–
	Dessert	–	–	100·0	100·0–125·0	–
	Total	506·0	462·0–607·8	498·6	427·7–518·6	0·478
Plate waste (%)	Bread	35·7	32·5–40·5	44·8	35·3–53·5	0·149
	Starchy food	16·5	13·6–24·7	32·5	25·3–40·2	<0·001
	Protein dish	14·5	12·4–18·1	33·5	22·1–43·3	<0·001
	Vegetables	34·9	32·0–50·7	52·9	41·5–70·1	0·005
	Fruit	26·2	15·8–40·0	55·5	41·8–64·2	0·002
	Other	22·3	17·6–27·0	11·8	11·3–12·3	–
	Dessert	–	–	14·9	13·2–19·0	–
	Total	23·7	21·5–26·8	41·5	33·2–42·6	<0·001
Plate waste/child (g)	Bread	9·4	5·1–14·9	12·3	9·8–15·1	1·000
	Starchy food	40·1	27·6–64·5	64·9	48·0–85·9	0·008
	Protein dish	9·4	5·1–15·9	19·1	13·5–35·5	0·002
	Vegetables	24·3	20·8–31·3	28·5	23·4–44·7	0·062
	Fruit	32·9	20·5–51·2	75·4	50·4–105·5	0·001
	Other	59·4	53·0–65·8	10·7	9·7–11·7	–
	Dessert	–	–	14·9	13·2–23·8	–
	Total	138·6	118·4–159·9	207·9	164·1–222·6	0·003

LOC-ORG, local-organic; ORG, organic.

*P* values refer to between-group comparison (LOC-ORG v. ORG), Mann–Whitney non-parametric test. Statistical analysis was not performed on the categories ‘Other’ and ‘Dessert’ due to limited number of data.

In the LOC-ORG case, the total waste per child accounted for a median of 138·6 g, with the highest contribution of the ‘other’ category (59·4 g as median), while in the ORG case it accounted for 207·9 g, with fruit contributing the most (75·4 g as median).

Beyond these findings, different trends of waste among dishes belonging to the same category were observed (see online supplementary material, Supplemental Tables S4–S7). For example, simple recipes among the starchy-based dishes (e.g. pasta or rice dressed with olive oil) were less wasted than more elaborate recipes (e.g. gnocchi with tomato sauce). Within the protein-based category, higher waste proportions were observed in the LOC-ORG when legumes or fish fillet were served, while in the ORG case both fish and cheese products were highly wasted (see online supplementary material, Supplemental Tables S4–S7).

### Nutritional impact of plate waste

Children attending the ORG schools were offered dishes with a significantly lower content of proteins (*P* < 0·05) and dietary fibre (*P* < 0·01), generating a waste higher in energy (*P* < 0·001), proteins (*P* < 0·05), carbohydrates (*P* < 0·01), soluble sugars (*P* < 0·05) and fat (*P* < 0·01). In parallel, the actual intake of energy (*P* < 0·01), proteins (*P* < 0·001), carbohydrates (*P* = 0·05) and dietary fibre (*P* < 0·001) was lower in the ORG case (Table 3).

**Table 3:** Macronutrient composition and fibre content of served lunch menus, of plate waste and of actual intake in the LOC-ORG (*n* 20) and ORG model (*n* 19)

		LOC-ORG		ORG		*P* value
		Mean	sd	Mean	sd	
Served lunches	Energy (kcal)	705·5	91·4	690·6	73·6	0·580
	Proteins (g)	30·5	5·9	26·9	4·7	0·041
	Carbohydrates (g)	104·1	12·1	103·6	13·3	0·899
	Soluble sugars (g)	22·6	6·5	22·4	8·4	0·929
	Dietary fibre (g)	12·9	3·5	9·4	3·3	0·002
	Fat (g)	20·4	7·1	20·8	4·6	0·829
	SFA (g)	7·0	6·4	5·8	3·2	0·432
	Cholesterol (mg)	49·8	34·4	68·2	57·8	0·230
Plate waste	Energy (kcal)	184·3	39·2	248·6	55·4	<0·001
	Proteins (g)	6·9	1·9	9·1	3·1	0·012
	Carbohydrates (g)	28·5	6·0	37·3	8·5	0·001
	Soluble sugars (g)	6·8	3·6	9·5	4·4	0·040
	Dietary fibre (g)	3·9	1·4	4·1	1·7	0·725
	Fat (g)	5·2	1·9	7·7	2·8	0·002
	SFA (g)	1·4	1·0	2·1	1·5	0·086
	Cholesterol (mg)	8·8	7·3	23·8	29·1	0·031
Actual intake	Energy (kcal)	521·2	69·8	442·0	79·5	0·002
	Proteins (g)	23·6	4·8	17·8	4·1	<0·000
	Carbohydrates (g)	75·6	10·3	66·2	13·5	0·018
	Soluble sugars (g)	15·7	5·6	12·8	7·5	0·175
	Dietary fibre (g)	9·0	2·7	5·3	2·0	<0·001
	Fat (g)	15·2	5·8	13·1	3·5	0·186
	SFA (g)	5·6	5·5	3·7	2·0	0·145
	Cholesterol (mg)	41·0	27·8	44·5	34·6	0·731

LOC-ORG, local-organic; ORG, organic.

*P* values refer to between-group comparison (LOC-ORG v. ORG), parametric *t* test.

As displayed in Table 4, the LOC-ORG model provided a higher content of some micronutrients in the served lunch. Accordingly, these differences and the different rate of plate waste reflected a discrepancy in the actual intake of vitamin C, K, P, Fe, and in the waste of vitamins B_1_, B_2_, B_3_, B_6_, and Zn.

**Table 4:** Micronutrient composition of served lunches, plate waste and actual intake in the LOC-ORG (*n* 20) and ORG model (*n* 19)

	*Vitamins*	LOC-ORG		ORG		*P*-value	*Minerals*	LOC-ORG		ORG		*P*-value
		Median	IR	Median	IR			Median	IR	Median	IR	
Served lunches	A (mg RE)	653·3	436·8–1385·6	470·4	312·3–751·1	0·107	Na (mg)	585·6	506·8–768·9	521·9	447·3–864·9	0·411
	B_1_ (mg)	0·5	0·4–0·6	0·4	0·4–0·5	0·396	K (mg)	1275·7	1051·9–1378·8	1050·1	807·9–1292·4	0·030
	B_2_ (mg)	0·4	0·3–0·5	0·4	0·3–0·4	0·627	Ca (mg)	279·7	261·2–321·5	258·0	193·9–314·1	0·113
	B_3_ (mg)	4·5	3·7–6·8	5·1	3·9–6·7	0·857	P (mg)	482·0	440·2–543·7	424·2	377·6–479·1	0·006
	B_6_ (mg)	0·7	0·6–0·8	0·8	0·6–0·9	0·270	Mg (mg)	42·9	26·0–63·6	31·9	29·0–55·1	0·708
	B_9_ (*μ*g)	108·7	92·1–142·7	100·7	69·7–121·4	0·113	Fe (mg)	4·8	4·3–5·9	3·9	3·6–4·6	0·007
	B_12_ (*μ*g)	1·3	0·4–1·6	1·2	0·9–1·8	0·879	Zn (mg)	3·4	2·6–3·9	3·1	2·7–3·9	0·857
	C (mg)	82·0	56·9–106·3	48·9	21·7–72·8	0·002	Cu (mg)	0·3	0·1–0·5	0·2	0·1–0·5	0·967
	D (*μ*g)	0·2	0·1–0·4	0·2	0·1–0·6	0·550						
Plate waste	A (mg RE)	236·3	93·5–418·8	226·5	110·6–6·9	0·813	Na (mg)	169·3	155·7–192·9	231·2	165·2–274·9	0·061
	B_1_ (mg)	0·1	0·1–0·2	0·2	0·1–0·2	0·006	K (mg)	343·9	275·2–411·0	425·9	346·8–456·4	0·134
	B_2_ (mg)	0·1	0·1–0·1	0·1	0·1–0·1	0·047	Ca (mg)	84·2	73·7–103·4	100·2	72·2–146·5	0·351
	B_3_ (mg)	1·0	1·0–1·2	1·7	1·4–2·2	0·004	P (mg)	115·3	102·3–140·4	149·8	111·9–174·6	0·050
	B_6_ (mg)	0·2	0·1–0·2	0·3	0·2–0·3	0·001	Mg (mg)	9·8	7·6–14·2	12·9	10·0–17·4	0·074
	B_9_ (*μ*g)	33·3	25·1–43·0	42·0	23·7–52·5	0·496	Fe (mg)	1·5	1·2–1·6	1·5	1·1–2·0	0·569
	B_12_ (*μ*g)	0·2	0·1–0·3	0·3	0·2–0·5	0·089	Zn (mg)	0·7	0·6–0·8	1·1	0·8–1·3	<0·001
	C (mg)	21·9	15·0–38·0	15·3	10·0–33·8	0·351	Cu (mg)	0·1	0·0–0·1	0·1	0·0–0·1	0·074
	D (*μ*g)	0·0	0·0–0·1	0·1	0·0–0·1	0·061						
Actual intake	A (mg RE)	406·7	316·0–939·2	244·7	167·3–375·6	0·006	Na (mg)	431·5	347·0–528·8	307·8	238·6–532·2	0·050
	B_1_ (mg)	0·3	0·3–0·4	0·3	0·2–0·3	0·015	K (mg)	908·7	770·0–1045·6	652·3	490·3–775·9	0·001
	B_2_ (mg)	0·3	0·2–0·4	0·2	0·2–0·3	0·033	Ca (mg)	205·2	177·8–231·0	158·1	104·3–206·7	0·011
	B_3_ (mg)	3·5	2·8–4·8	3·2	2·2–4·5	0·270	P (mg)	380·3	349·7–422·0	285·6	221·5–297·5	<0·001
	B_6_ (mg)	0·5	0·4–0·6	0·5	0·5–0·6	0·879	Mg (mg)	33·3	19·6–46·2	23·7	16·5–37·1	0·184
	B_9_ (*μ*g)	77·1	63·4–99·7	50·6	38·2–63·6	0·002	Fe (mg)	3·6	2·8–4·2	2·4	2·0–2·9	<0·001
	B_12_ (*μ*g)	1·1	0·3–1·3	0·8	0·6–1·1	0·351	Zn (mg)	2·6	2·1–3·1	2·0	1·8–2·7	0·084
	C (mg)	53·7	35·6–63·6	19·8	13·1–33·2	<0·001	Cu (mg)	0·2	0·1–0·4	0·1	0·1–0·3	0·728
	D (*μ*g)	0·2	0·1–0·3	0·2	0·1–0·4	0·857						

LOC-ORG, local-organic; ORG, organic.

*P* values refer to between-group comparison (LOC-ORG *v*. ORG), Mann–Whitney non-parametric test.

**Fig. 1:**
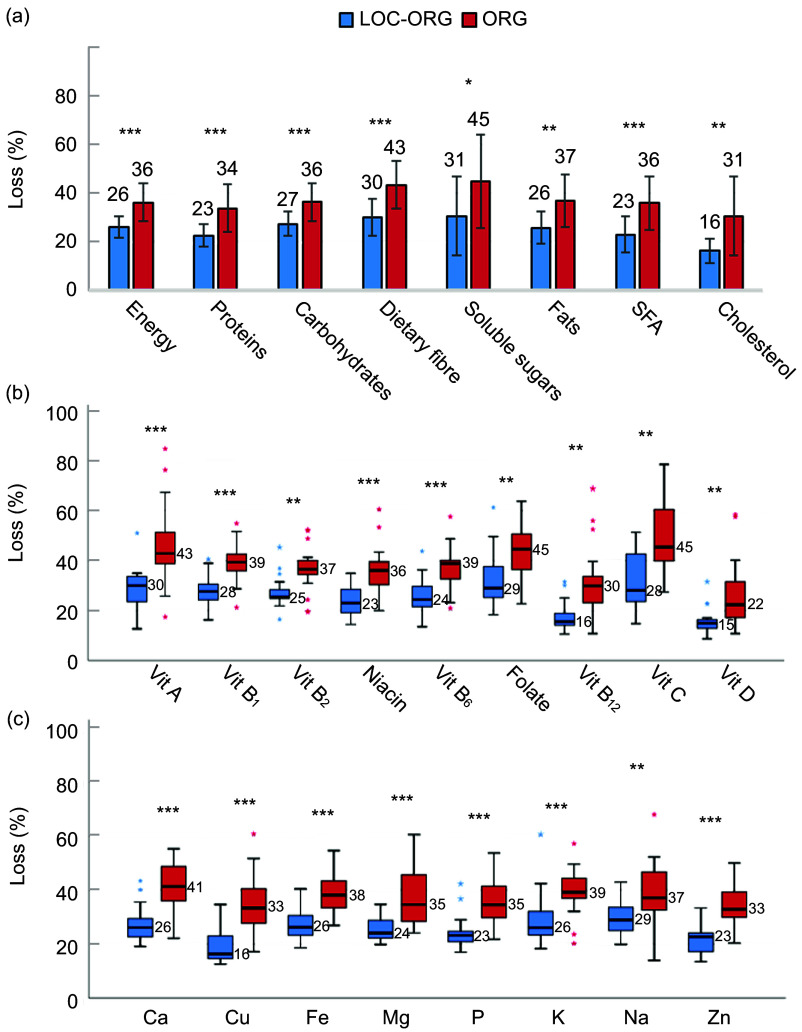
Losses of energy and nutrients of school lunches in the LOC-ORG (*n* 20) and ORG model (*n* 19). *Note*: Losses of energy and macronutrients (a); vitamins (b); and minerals (c) of school lunches in the LOC-ORG (*n* 20) and ORG model (*n* 19). Data are expressed as mean ± sd for a, and median (IR) for b and c. CHO: carbohydrate. Parametric *t* test (a) and Mann–Whitney non-parametric test (b, c). *P < 0·05; **P < 0·01; ***P < 0·001. LOC-ORG, local-organic; ORG, organic

By adjusting for energy and nutrient amounts of plate waste, children in the LOC-ORG schools consumed a mean of 74 % of the total energy of the offered lunch, accounting for 10 % more than children in the ORG schools. Both energy and nutritional losses were significantly higher in the ORG case compared with the counterpart (Fig. 1(a)).

In terms of macronutrients, the mean losses ranged from 16 % to 31 % in the LOC-ORG schools and from 31 % to 45 % in the ORG schools, polarising the loss of cholesterol and soluble sugars. In accordance with the high plate waste of vegetables, dietary fibre was on average highly wasted in both the cases (30 %, LOC-ORG and 43 %, ORG).

The micronutrient losses for vitamins ranged from a median of 15 % (vitamin D) to 30 % (vitamin A) in the LOC-ORG case and from 22 % (vitamin D) to 45 % (vitamin B_9_ and C) in the ORG model (Fig. 1(b)). For vitamin B_12_, a relatively high intake was registered, resulting in lower losses, with respectively a median of 16 % and 30 % in the LOC-ORG and ORG. Among minerals, the median losses ranged from 16 % (Cu) to 29 % (Na) in LOC-ORG schools and from 33 % (Cu and Zn) to 41 % (Ca) for the ORG ones (Fig. 1(c)).

When compared with the national reference, the energy and nutrient distribution of the school menus considerably changed after subtraction of the plate waste (see online supplementary material, Supplemental Figures S1 and S2). Although the proportion of the energy provided by total proteins, fats and carbohydrates did not substantially differ, only 50 % of the LOC-ORG lunches and 21 % of the ORG menus reached the minimum energy threshold. The loss of fat content was the most severe, with 5 % of LOC-ORG lunches and 11 % of ORG lunches having adequate values, while the protein content had the best outcomes, with all the LOC-ORG menus and 84 % of the ORG menus being compliant with the recommendations.

### Carbon impact of plate waste

In LOC-ORG model, food production accounted for 95 % of the total GHGe linked to plate waste (Table 5) and corresponded to 19 % of the total carbon footprint due to the total food supplied by the school catering service during the data collection days, estimated to be 3991 kgCO_2_eq. Similarly, in the ORG case, plate waste GHGe related to food production were 92 % of the total plate waste carbon footprint (Table 5) and represented 28 % of the total GHGe due to the food supplied by the catering service during the data collection days, estimated to be 2790 kg CO_2_eq. The food waste impact for the average lunch meal served to children was 0·2 kg CO_2_eq for the LOC-ORG and 0·3 kg CO_2_eq for the ORG model, corresponding respectively to a share of 20 % and 31 %.

**Table 5:** Greenhouse gas emissions (kg CO_2_eq) and average emissions factors (kg CO_2_eq/kg) estimated for plate waste and serving in LOC-ORG and ORG cases considering the contribution of each food category and the contribution of food production, transportation, and waste handling

	LOC-ORG		ORG		LOC-ORG	ORG	LOC-ORG	ORG
	kg CO_2_eq	%	kg CO_2_eq	%	kg CO_2_eq/kg	kg CO_2_eq/kg	g CO_2_eq/serving	g CO_2_eq/serving
Production	761·9	94·8	792·5	92·3	1·41	1·44	322·3	344·1
Starchy food	414·4	51·6	402·1	46·8	1·92	1·78	110·3*	133·9*
Protein dish	139·5	17·4	265·3	30·9	3·54	4·41	40·2	105·2
Vegetables	60·9	7·6	23·4	2·7	0·62	0·34	15·3	16·1
Fruit	95·6	11·9	62·9	7·3	0·58	0·35	23·1	27·3
Dessert	–		35·9	4·2	–	2·61	–	52·0
Other	51·6	6·4	3·0	0·3	2·28	0·92	133·3	9·6
Transportation	15·5	1·9	38·4	4·5	0·03	0·07	0·8	0·1
Waste handling	25·9	3·2	27·4	3·2	0·05	0·05	1·3	0·2
Total	803·3		858·3		1·49	1·56	324·4	344·5

LOC-ORG, local-organic; ORG, organic.

Transportation refers to the distance between the central kitchen and the schools.

* To compute the average emission factors per serving of starchy food, the average number of servings calculated between the starchy food and bread category for the two case studies (i.e. *n* 3757, LOC-ORG and *n* 3004, ORG) has been applied.

Considering the contribution of single wasted food categories to the total carbon footprint, starchy food was responsible for 52 % (LOC-ORG case) and 47 % (ORG case), followed by protein-based dishes that exhibited a share of 17 % (LOC-ORG) and 31 % (ORG).

In particular, the food items that contributed more within the protein-based plates to the GHGe were meat and fish (50 %) in the ORG case, and soft and hard cheese (39 %) (see online supplementary material, Supplemental Table S8). Conversely, fruit and vegetables represented together about 19 % (LOC-ORG) and 10 % (ORG) of the total carbon burden, although they accounted for more than 45 % of the total food waste; the food included within the categories less represented in the school menus impacted respectively from < 1 % (ORG) to 6 % (LOC-ORG) when considering ‘other’ food and 4 % when considering dessert.

In both models, the transportation of food destined to be wasted had a marginal carbon impact (2 % and 4·5 % of the total food waste emission, in LOC-ORG and ORG, respectively). Similarly, the impact of food waste management was very low (3 % in both models), which was based on the composting system.

In both cases, the wasted protein dishes show relatively higher emissions factors (3·54 kg CO_2_eq/kg, LOC ORG; 4·41 kg CO_2_eq/kg, ORG) followed by the ‘other’ category for the LOC-ORG case (2·28 kg CO_2_eq/kg) and by dessert for the ORG case (2·61 kg CO_2_eq/kg). The GHGe associated with kg of wasted fruit and vegetables were instead the lowest (0·58 kg CO_2_eq/kg and 0·62 kg CO_2_eq/kg, LOC ORG; 0·35 kg CO_2_eq/kg and 0·34 kg CO_2_eq/kg, ORG). When GHGe are considered per serving, the most impactful categories were instead ‘other’ (133·3 g CO_2_eq/g) followed by starchy food (110·3 g CO_2_eq/g) for the LOC-ORG case and starchy food (133·9 g CO_2_eq/g) followed by protein dishes (105·2 g CO_2_eq/g) for the ORG case. Overall, on average, the meal served in the LOC-ORG case had a lower carbon footprint compared with the counterpart (324·4 g CO_2_eq/g *v.* 344·5 g CO_2_eq/g).

### Economic impact of plate waste

The plate waste collected respectively in the LOC-ORG and ORG cases corresponded to a total cost of € 978 and € 1462, equivalent to € 1·81 and € 2·65 per kg of waste (Table 6). Therefore, the cost associated with the total daily plate waste collected in the two case studies is € 48·9 and € 77·0 in LOC-ORG and ORG models, respectively.

**Table 6:** Economic impact estimated for plate waste and serving in the LOC-ORG and ORG cases

Food category	Cost (€)		Average cost (€/kg)		Average cost (€/serving)	
	LOC-ORG	ORG	LOC-ORG	ORG	LOC-ORG	ORG
Starchy food	427	653	1·98	2·89	0·11*	0·22*
Protein dish	237	370	6·00	6·15	0·07	0·15
Vegetables	134	110	1·36	1·61	0·03	0·08
Fruit	157	218	0·96	1·21	0·04	0·09
Dessert	–	90	–	6·57		0·13
Other	24	22	1·07	6·57	0·06	0·07
Total	978	1·462	1·81	2·65	0·24†	0·48†

LOC-ORG, local-organic; ORG, organic.

* To compute the average cost per serving of starchy food, the average number of servings calculated between the starchy food and bread category for the two case studies (i.e. *n* 3757, LOC-ORG and *n* 3004, ORG) has been applied.

† The economic cost refers to the average meal.

Starchy food contributed the most to the total plate waste economic cost both in the LOC-ORG case (44 %) and in the ORG case (45 %). In this category, bread consistently contributed accounting for 24 % and 17 % of the food waste cost in the LOC-ORG and ORG case, respectively. Altogether, fruit and vegetables accounted for 30 % (LOC-ORG) and 22 % (ORG) of the total plate waste cost and contributed about 50 % to the total waste. Protein dishes, together with the ‘other’ category, showed a share of 27 % of the total economic loss in both the case studies, while desserts impacted for the remaining 6 % in the ORG case.

Considered singularly, in LOC-ORG case, protein-based plates accounted for more than 20 % of the total food waste cost, although they entailed less than 11 % of the total waste amount. Within the plant-based food category, the most expensive food item was the codfish for the LOC-ORG case (48 %) and fresh cheese, accounting for 35 % of the total cost of the same food category, followed by turkey meat (13 %) and cured meat (11 %), for the ORG model (see online supplementary material, Supplemental Table S9).

The highest average cost per kg of wasted food in the ORG case can be attributed to dessert and the ‘other’ category (both 6·57 €/kg) followed by protein dishes (6·15 €/kg), which were the most expensive in the LOC-ORG case (6·00 €/kg). Similar to the environmental results, fruit and vegetables showed a relatively low impact, both considering their contribution to the total plate waste cost and the average cost per kg of wasted food category. However, the estimated cost of plate waste per meal was double in the ORG model (0·48 €/meal *v*. 0·24 €/meal). In relative terms, the estimated cost of plate waste represents 3·9 % and 9·6 % of the full price paid by parents in the LOC-ORG and ORG models, respectively. Among food categories, when the average cost per serving is considered, the most expensive was starchy food for both the case studies. According to the estimates, for ORG model the total economic loss associated with plate waste as a proportion of the total food procurement cost was 32 %, while for LOC-ORG model the plate waste cost accounted for 21 % of the meal service budget referred to the 2017–2018 school year.

## Discussion

In this study, primary schoolchildren’s plate waste was quantified, and its nutritional, environmental, and economic implications were estimated. Two food procurement models (local-organic and organic) were considered and compared, with the LOC-ORG model showing a lower waste. Across food categories, vegetables and fruit were highly wasted, followed by bread. The waste of vegetables and fruit reached relatively high proportions, mainly in the ORG case, where pupils’ intake was, as median, less than half of the serving size offered to them. Vegetables were however highly discarded in all schools. Conversely, the protein-based dishes in the LOC-ORG case model and pizza in the ORG case registered the lowest waste percentages. Among starchy food dishes, simple recipes reported relatively lower plate waste compared with more complex recipes; nevertheless, due to the limited number of observations, it is not possible to derive a definitive picture from these results. Starchy food (including bread) accounted for the highest proportions against the total food waste collected in both the case studies (about 40 %), similarly to what was found in a Chinese study^(37)^ for staple food (43 %). The authors reported however a higher share for vegetables (42 %) compared with what found in this study (18 % LOC-ORG and 12 % ORG).

The plate waste percentage found for vegetables in the LOC-ORG case equals that reported by Boschini and colleagues^(38)^ for side dishes in a study focusing on the Italian primary school context. The authors reported instead a significantly lower plate waste for bread (8 %), either compared with the LOC-ORG or ORG case.

From the analysis of the nutritional consequences of plate waste, a higher detrimental impact was reported for the ORG model compared with the LOC-ORG one, in which on average, compared with the meal offered at school, the loss of energy and macronutrients was approximately 10 % lower than in the ORG model. In accordance with plate waste data, soluble sugars and dietary fibre presented the highest shares of losses. Among micronutrients, the limited loss of vitamin B_12_ converges with the preferential consumption of protein-based foods by children compared with other food categories. On the contrary, because of the consistent waste of fruit and vegetables, vitamin C and folate were highly lost (from 28 % to 45 %) across the case studies.

When adjusted for plate waste data, at least half of the sampled lunch meals fell below the national energy recommendations, and a wide range of the school lunches did not reach the national standards. In terms of compliance with the national reference values, Dinis and colleagues^(39)^ found a lower share of lunches being adequate compared with LOC-ORG or ORG. Their study was carried out in Portugal, where primary schools have narrower national energy and nutritional standards compared with the Italian ones. Their plate waste analysis showed relatively higher percentages for vegetables (>60 %), while fruit was discarded at a lower rate (24 %) than in Italy. On the other hand, comparable results can be observed for starchy-based dishes for which the waste was similar (44 %, males; and 47 %, female) to the ORG case^(39)^. Among protein-based dishes, meat dishes were wasted in lower proportions compared with fish dishes. This pattern was observed both in Portuguese children (31 % and 32 % *v.* 55 % and 58 %, respectively, in males and females)^(39)^ and in the two Italian case studies (on average 11 %, LOC-ORG and 25 %, ORG *v*. 17 %, LOC-ORG and 32 %, ORG).

With regard to the environmental impact of plate waste, starchy food greatly impacted in both the case studies in similar proportions, while for protein-based dishes a different pattern is suggested, with a higher share in the ORG case. The different composition of the food waste explains the associated carbon footprint being 5 % higher in the ORG compared with the counterpart. In a similar study evaluating the carbon footprint of food waste generated in nursery and primary public schools of Cento (Italy), the Global Warming Potential of food waste was estimated to be 15–18 % of the total meal impact^(20)^. It was below the percentages of the carbon emissions embedded in the meal waste reported in the present study (20–31 %). The composition of food waste can explain such discrepancy, as in the present study, the starchy food contributes at most to the total plate waste in the two case studies, while in the Cento’s study, vegetables had a relatively higher contribution.

Concerning the economic perspective, the loss cost per kg of food waste was 43 % higher in the ORG than in the counterpart. For families the plate waste cost impacts the budget spent for the service, with a share from 4 % (LOC-ORG model) to 10 % (ORG model) of the full price paid per lunch meal. The economic value of plate waste represents a significant share of the total school meals service budget too, with about one-fifth (LOC-ORG case) and one-third (ORG case) of the budget for the food procurement spent on food that will be discarded by children. These findings are consistent with a study carried out in Cento, in which the economic impact of food waste is estimated in a range of 6–26 % compared with the total meal cost^(20)^.

### Hypothetical plate waste determinants

The higher occurrence of more familiar local/traditional quality products (i.e. mainly PDO cheese and cured meat products) in the LOC-ORG model could have contributed to determine different plate waste scenarios. However, a multitude of individual, social and environmental factors, including meal recipes^(40)^, food texture^(40,41)^, food preference^(42,43)^, the canteen environment^(44)^ and teacher engagement^(45,46)^, can exert an influence. Surprisingly, cooking in an onsite kitchen has been found to determine higher plate waste compared with cooking in an offsite kitchen^(46)^. Indeed, we can expect that the transport could negatively modify the sensory characteristics of cooked food (e.g. food texture and temperature at which food is served) at the time of consumption. However, we do not have supportive data from our study to substantiate this hypothesis. Further investigations in this direction are warranted. Furthermore, only in the Parma school canteens (LOC-ORG), the quantification of plate waste was performed by the caterer every month to optimise the meal planning, preparation and distribution. In this occasion, children had to separate their leftover into dedicated bins. A different scenario was observed in Lucca (ORG model), where only older students, after having lunch in one of the two schools, were used to contribute to clear the tables. These findings suggest the importance of school catering management and organisation and schoolteachers’ commitment in driving children towards more sustainable eating behaviours and food-related habits. Indeed, dealing with the food waste issue in classrooms has shown a positive influence on children’s attitudes, knowledge and behaviour^(47)^.

### Strengths and limitations

To quantify plate waste, the gold standard technique (i.e. the weighting method) was applied. Moreover, a wide range of LCA emission factors was adopted to mitigate the uncertainty level in the environmental results and to capture the specificity of the production processes (local raw materials and methods of production) of the local/quality foods served in the Italian school canteens. Whereas, for market food prices a short-run perspective was applied using average yearly prices. However, important limitations should be recognised. First, the data collection was performed in a few schools and the sampled menus cover only a proportion of the total offer. Therefore, the representativeness of our findings is not guaranteed in other organic and local-organic procurement models in Italy. Secondly, the nutritional analysis cannot rely on validated national nutritional databases focusing on organic products. Consequently, the nutritional evaluation of school menus did not consider possible discrepancies in the nutritional content of organic products compared with the conventionally grown food. Considering the environmental impact, beside the carbon footprint, a wider set of environmental indicators could have been considered, for example, human toxicity, eco-toxicity, biodiversity loss and animal welfare^(48)^. Last, due to the confidentiality content of the food procurement contracts between caterers and food suppliers, the present study estimates the economic impact of plate waste using the food prices available in national agri-food market survey datasets and not the actual price of each food item.

### Conclusion

The present study highlights relatively high percentages of plate waste in primary schools located in two Italian municipalities, with the highest proportions for vegetables and fruit responsible for major losses of soluble sugars, dietary fibre, vitamin C and folate. Environmental and economic implications of waste were instead particularly relevant for starchy food and protein-based dishes, although less discarded. To minimise the food discarded by children at school, both the municipalities and the caterers need to identify the contextual determinants and develop effective strategies accounting for the school governance and catering management. Furthermore, the technical specifications of the school meals service procurement contracts could be directed to strengthen the commitment of the school meals service supply chain to develop new methods/techniques of meal design and preparation. In addition, nutritional and environmental education should be integrated into primary school programmes to increase the awareness on food waste impacts in children and teachers. More specifically, among the virtuous actions addressing the need to simultaneously minimise nutritional, economic and environmental plate waste implications^(49)^, catering managers should recognise the contribution of the meals service and staff to children’s education by rewarding the ability of the catering staff to increase meal uptake thanks to high-quality interaction and supervision, facilitate engagement with pupils and parents in menu design and planning, find strategies to increase fruit and vegetable consumption through menu development, support an improved canteen design with a fun layout, and ensure to allow a proper time for eating lunch and to serve adequate portion sizes for age and appetite. With the aim to reduce plate waste, a virtuous implementation of these strategies, together with collecting waste monitoring practices, could compensate a procurement model with a lower share of local/traditional products, whose setting is rather static, as defined by the procurement contract.

## Supporting information

Biasini et al. supplementary material Biasini et al. supplementary material

## Supplementary material

For supplementary material accompanying this paper visit https://doi.org/10.1017/S136898002400034X.
